# Modification of Xyloglucan Metabolism during a Decrease in Cell Wall Extensibility in 1-Aminocyclopropane-1-Carboxylic Acid-Treated Azuki Bean Epicotyls

**DOI:** 10.3390/plants12020367

**Published:** 2023-01-12

**Authors:** Aya Yamaguchi, Kouichi Soga, Kazuyuki Wakabayashi, Takayuki Hoson

**Affiliations:** 1 Department of Biology, Graduate School of Science, Osaka City University, Sumiyoshi-ku, Osaka 558-8585, Japan; 2 Department of Biology, Graduate School of Science, Osaka Metropolitan University, Sumiyoshi-ku, Osaka 558-8585, Japan

**Keywords:** azuki bean (*Vigna angularis*), cell wall extensibility, ethylene, growth inhibition, molecular mass, xyloglucans

## Abstract

The exogenous application of ethylene or 1-aminocyclopropane-1-carboxylic acid (ACC), the biosynthetic precursor for ethylene, to plants decreases the capacity of the cell wall to extend, thereby inhibiting stem elongation. In this study, the mechanism by which the extensibility of cell walls decreases in ACC-treated azuki bean epicotyls was studied. ACC decreased the total extensibility of cell walls, and such a decrease was due to the decrease in irreversible extensibility. ACC increased the molecular mass of xyloglucans but decreased the activity of xyloglucan-degrading enzymes. The expression of *VaXTHS4*, which only exhibits hydrolase activity toward xyloglucans, was downregulated by ACC treatment, whereas that of *VaXTH1* or *VaXTH2*, which exhibits only transglucosylase activity toward xyloglucans, was not affected by ACC treatment. The suppression of xyloglucan-degrading activity by downregulating *VaXTHS4* expression may be responsible for the increase in the molecular mass of xyloglucan. Our results suggest that the modification of xyloglucan metabolism is necessary to decrease cell wall extensibility, thereby inhibiting the elongation growth of epicotyls in ACC-treated azuki bean seedlings.

## 1. Introduction

Ethylene is a gaseous plant hormone that has significant effects on plant growth and development [1]. The inhibition of elongation growth and promotion of lateral expansion of stems have been recognized as principal ethylene effects in plants. 1-Aminocyclopropane-1-carboxylic acid (ACC) is the immediate precursor of ethylene [2]. The production of ethylene induces ethylene responses such as the inhibition of elongation growth and promotion of lateral expansion of stems, when seedlings are treated with ACC. Therefore, ACC has been widely used to analyze the effects of ethylene on plant growth and development.

The capacity of the cell wall to extend (extensibility of cell walls) determines the rate of elongation growth of individual cells; therefore, it regulates the elongation growth of the whole plant [3]. Ridge (1973) [4] reported that ethylene decreased the extensibility of cell walls, which was determined by a plasmolytic method during the inhibition of elongation growth of pea epicotyls. In addition, Wu et al. (2020) [5] reported that ACC increased cell wall stiffness, which was measured by nanoindentation atomic force microscopy during the inhibition of elongation growth of Arabidopsis hypocotyls. Recently, we analyzed the effect of ACC on cell wall extensibility by load–extension analysis using a tensile tester and found that ACC decreased cell wall extensibility during the inhibition of elongation growth of azuki bean (*Vigna angularis*) epicotyls. It is indicated by these results that the decrease in the extensibility of cell walls caused by ethylene leads to the inhibition of elongation growth of stems. However, the mechanism by which ethylene decreases the extensibility of cell walls remains unclear.

The primary cell walls of flowering plants consist of cellulose microfibrils tethered by hemicellulosic polysaccharides and embedded in pectic polysaccharides [6]. In flowering plants with a Type I cell wall, xyloglucans are predominant hemicellulosic polysaccharides, and the modification of the molecular mass of xyloglucans is required to regulate the extensibility of cell walls. For example, auxin increases the extensibility of cell walls by decreasing the molecular mass of xyloglucans, thereby stimulating the elongation growth of azuki bean epicotyls [7]. On the contrary, light irradiation to etiolated pea epicotyls decreased the extensibility of cell walls by increasing the molecular mass of xyloglucans, thereby inhibiting elongation growth [8]. In addition, hypergravity, gravitational force more than 1 *g*, induced similar changes in the molecular mass of xyloglucans and the extensibility of cell walls when it suppressed elongation growth in epicotyls of azuki bean [9]. It is suggested by these findings that ethylene increases the molecular mass of xyloglucans in flowering plants with a Type I cell wall, thereby decreasing the extensibility of cell walls.

Xyloglucan endotransglucosylase/hydrolases (XTHs) endolytically cleave xyloglucan polymers and join the newly generated end to another xyloglucan chain (transglucosylase activity) or to H_2_O (hydrolytic activity) [10,11]. The metabolism of xyloglucans has been studied vigorously in azuki bean epicotyls. Three azuki bean (*Vigna angularis*) XTHs (VaXTH1, VaXTH2, and VaXTHS4) have been identified. VaXTH1 and VaXTH2 only exhibit transglucosylase activity, but not hydrolytic activity toward xyloglucans [12]. On the contrary, VaXTHS4 only exhibits hydrolytic activity toward xyloglucans, but it does not show transglucosylase activity [13]. The expression of *VaXTHS4* was downregulated when hypergravity increased the molecular mass of xyloglucans [14]. However, neither the expression of *VaXTH1* nor that of *VaXTH2* were affected by hypergravity. It is indicated by these findings that the suppression of xyloglucan-degrading activity because of the downregulation of *VaXTHS4* expression increases the molecular mass of xyloglucans in hypergravity-treated azuki bean epicotyls. Maurice et al. (1981) [15] reported that ethylene reduced xylose and glucose levels in the cell wall solution collected from ethylene-treated segments of pea epicotyls, and proposed that ethylene suppresses degradation of xyloglucans. Collectively, these findings suggest that in flowering plants with a Type I cell wall, ethylene increases the molecular mass of xyloglucans by decreasing xyloglucan-degrading activity, which decreases the extensibility of cell walls, thereby inhibiting elongation growth of stems.

This study aimed to determine the mechanism by which ACC treatment of azuki bean seedlings decreases the cell wall extensibility. We investigated the effects of ACC on the molecular mass of xyloglucans, xyloglucan-degrading activities, and expression of *XTH*s. Our results show that the suppression of xyloglucan-degrading activity by downregulating *VaXTHS4* expression may be necessary to increase the molecular mass of xyloglucans, and the modification of xyloglucan metabolism is necessary to decrease the extensibility of cell walls, thereby inhibiting elongation growth of stems. Ethylene is involved in the response to abiotic stresses such as heat, cold, and drought, which inhibit the growth of stems and reduce the yields of crops [16]. Thus, artificial modification of xyloglucan metabolism may relieve ethylene-induced decreases in the cell wall extensibility and relieve the abiotic stress-induced inhibition of plant growth.

## 2. Results

### 2.1. Growth and Extensibility of Cell Walls

**Figure 1:**
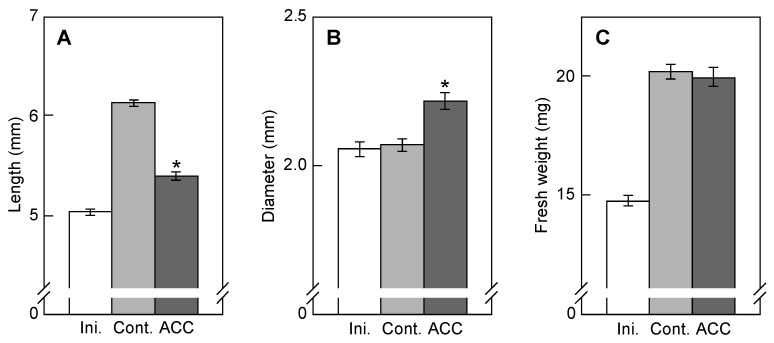
Length, diameter, and fresh weight of ACC-treated azuki bean epicotyls. ACC at 10^−5^ *M* was applied to seedlings marked with a 5-mm region on epicotyls for 5 h at 25 °C under dark conditions. The length (**A**), diameter (**B**), and fresh weight (**C**) of marked regions are shown. Values are presented as the means ± SE (*n* = 30). * *p* < 0.05, mean with significant differences between control and ACC treatments.

In 25–30-mm-long epicotyls, growth occurred only in the apical region [17]. Hence, the region 3–8 mm below the hook of epicotyls was analyzed in this study. The effects of ACC on elongation growth and lateral growth from 10^−7^ to 10^−5^ *M* were dose-dependent, but the effects began to saturate at 10^−4^ *M* [17]. Therefore, 10^−5^ *M* ACC was used in this study. The length, diameter, and fresh weight of the region with 10^−5^ *M* ACC for 5 h are shown in Figure 1; “Ini. (initial)” represents the result immediately before treatment (0 h), “Cont. (control)” represents the result without ACC treatment for 5 h, and “ACC” represents the result with ACC treatment for 5 h. The length increased during 5 h incubation with or without ACC, but ACC treatment significantly inhibited elongation; elongation of the epicotyls was reduced to about 30% of the control (Figure 1A). The diameter did not change during 5 h incubation without ACC, but ACC treatment significantly increased the diameter; the cross-sectional area calculated from the diameter of epicotyls increased by about 15% of the control (Figure 1B). The fresh weight increased during 5 h incubation with or without ACC, and ACC did not affect this increase (Figure 1C).

**Figure 2:**
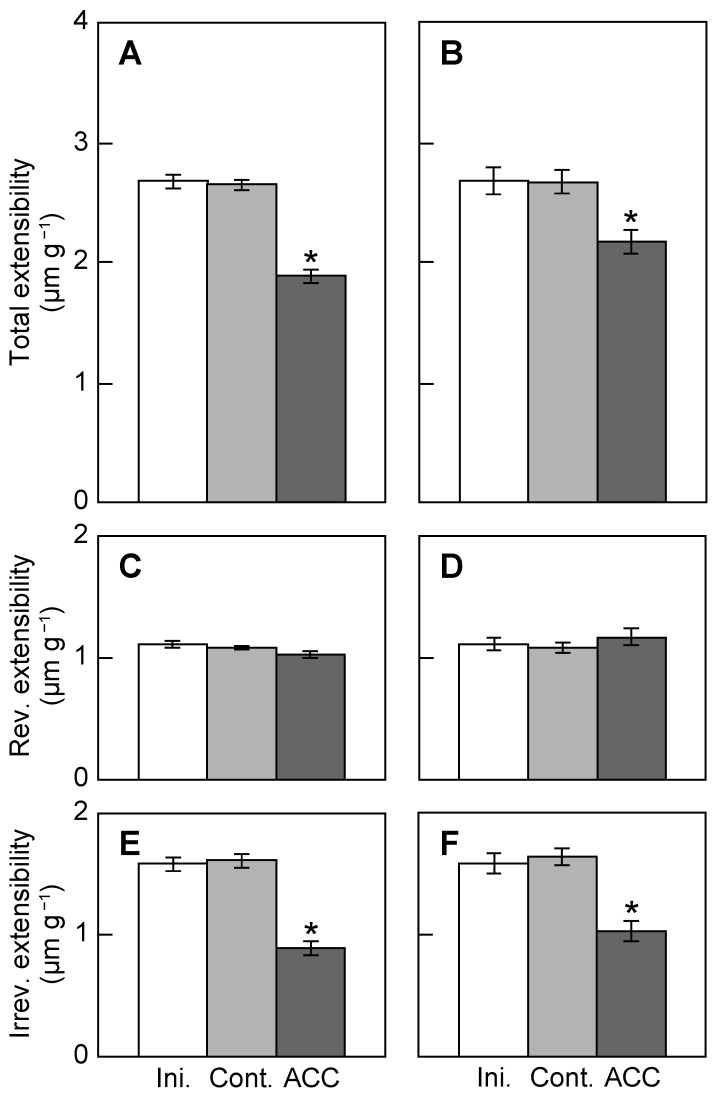
Total, reversible, and irreversible cell wall extensibility of ACC-treated azuki bean epicotyls. ACC treatment was carried out in accordance with the parameters shown in Figure 1. The material was extended two times consecutively using a tensile tester. Total extensibility (**A**) was determined by the slope of the load–extension curve at the first extension. Reversible extensibility (**C**) was determined by the slope of the second curve, and irreversible extensibility (**E**) was determined by the difference between the slopes of the first and second extensions. The total extensibility, reversible extensibility, and irreversible extensibility were normalized by multiplying the ratios of the cross-sectional area of epicotyls, wherein the area of the initial epicotyls was equal to one (**B**,**D**,**F**). Values are the means ± SE (*n* = 30). * *p* < 0.05, mean with significant differences between control and ACC treatments.

Figure 2A,C,E show the total extensibility, reversible extensibility, and irreversible extensibility, respectively, whereas Figure 2B,D,F show the results of Figure 2A,C,E normalized by multiplying the ratios of the cross-sectional area of epicotyls, wherein the area of the initial epicotyls was equal to one. The total extensibility did not change during 5 h incubation without ACC treatment (Figure 2A,B). However, the total extensibility with or without correction by the cross-sectional area was significantly decreased by ACC treatment, indicating that ACC reduces the capacity of the cell wall to extend in epicotyls. The total extensibility of epicotyls was divided into reversible and irreversible elements by two sequential extensions. ACC did not affect the reversible extensibility (Figure 2C,D). However, the irreversible extensibility of epicotyls with or without correction by the cross-sectional area was significantly decreased by ACC treatment (Figure 2E,F). Thus, it is indicated by these results that the decrease in the total extensibility of epicotyls by ACC treatment was primarily due to the decrease in irreversible extensibility.

### 2.2. Molecular Mass of Xyloglucans

**Figure 3:**
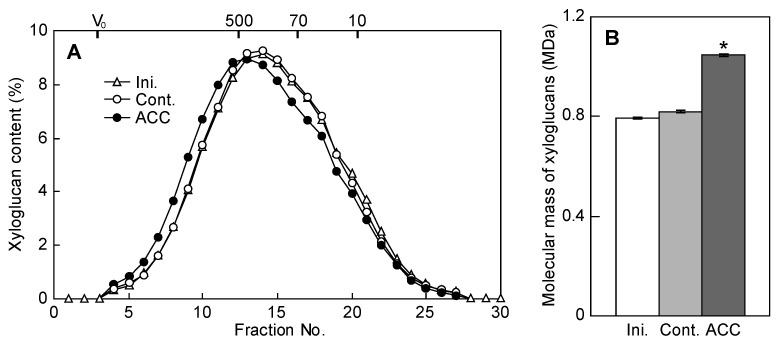
Molecular mass distribution and molecular mass of xyloglucans of ACC-treated azuki bean epicotyls. ACC treatment was carried out in accordance with the parameters shown in Figure 1. The elution profiles of xyloglucans (**A**) on a gel permeation column (TSK-GEL 5000 PW) are shown as the means of three replicates without SE. Vertical bars indicate the elution position of void volume (V_0_) and molecular mass standards (in kDa). The weight-average molecular mass of xyloglucans (**B**) was determined on the basis of the elution profile of xyloglucans. Values in (**B**) are presented as the means ± SE (*n* = 3). * *p* < 0.05, mean with significant differences between control and ACC treatments.

The molecular mass distribution of xyloglucans in the hemicellulose-II fraction is shown in Figure 3A. Xyloglucans of the initial and control epicotyls were eluted in regions with almost the same molecular weight, whereas those obtained from ACC-treated epicotyls shifted toward the high-molecular-mass regions. Thus, the calculated weight-average molecular mass of ACC-treated epicotyls was significantly higher than that of the xyloglucans in initial and control epicotyls (Figure 3B).

### 2.3. Activity of Xyloglucan-Degrading Enzymes and XTH Expression

**Figure 4:**
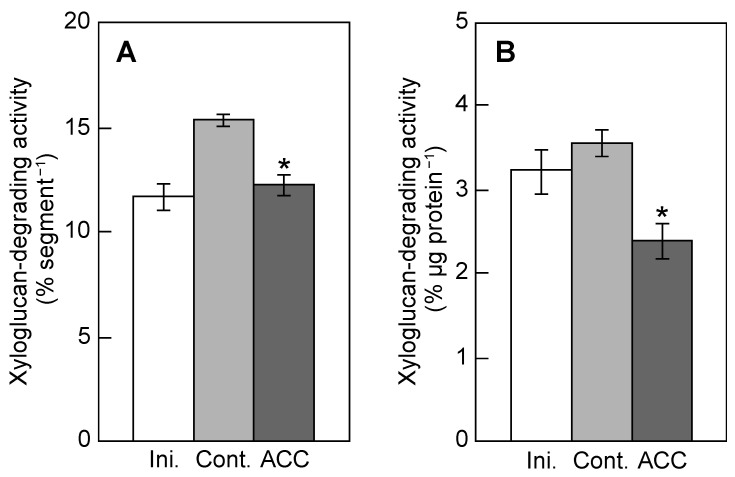
Activities of xyloglucan-degrading enzymes of ACC-treated azuki bean epicotyls. ACC treatment was carried out in accordance with the parameters shown in Figure 1. The activity of xyloglucan-degrading enzymes was measured by iodine staining, and indicated by the decrease in absorbance at 640 nm of the xyloglucan–iodine complex per segment (**A**) and per microgram of protein (**B**). Values are presented the means ± SE (*n* = 3). * *p* < 0.05, mean with significant differences between control and ACC treatments.

The xyloglucan-degrading activity in a protein fraction obtained from the cell walls is shown in Figure 4. The activity per segment (epicotyl region) increased during 5 h incubation in control epicotyls, but the increase was suppressed in ACC-treated epicotyls (Figure 4A). The activity per microgram of protein was similar in the initial and control epicotyls but was significantly reduced by ACC (Figure 4B).

**Figure 5:**
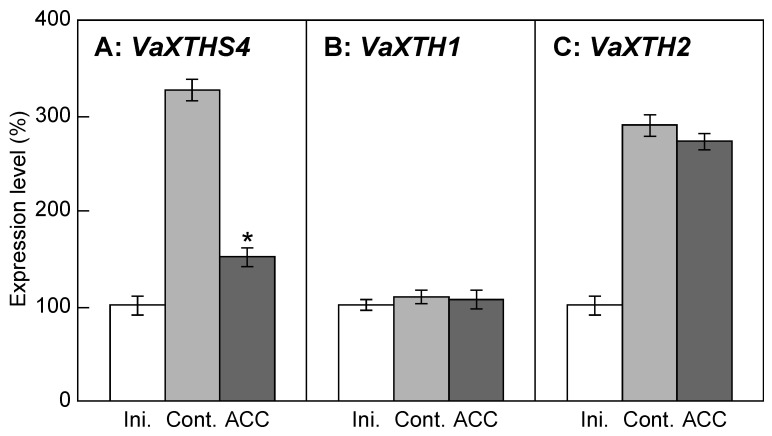
Gene expression of *XTH*s of ACC-treated azuki bean epicotyls. ACC treatment was carried out in accordance with the parameters shown in Figure 1. The gene expression of *VaXTHS4* (**A**), *VaXTH1* (**B**) and *VaXTH2* (**C**) was determined using a real time RT–PCR. The values were corrected for the levels of 18S rRNA as an internal standard and represented as the percentage of the initial levels. Values are presented as the means ± SE (*n* = 3). * *p* < 0.05, mean with significant differences between control and ACC treatments.

The effects of ACC on the expression level of *VaXTHS4*, *VaXTH1*, and *VaXTH2* in the epicotyl region were examined (Figure 5). *VaXTHS4* expression increased with or without ACC during 5 h incubation (Figure 5A). However, the increment of *VaXTHS4* expression in epicotyls treated with ACC was less than one-fifth that of the control. *VaXTH1* expression was nearly constant during 5 h incubation with or without ACC (Figure 5B). On the contrary, *VaXTH2* expression in control and ACC-treated epicotyls was increased to a certain extent during 5 h incubation (Figure 5C). It is indicated by these results that the expression of only *VaXTHS4* was controlled in response to ACC among the three *XTH* genes.

## 3. Discussion

Measurements of mechanical properties of cell walls using a plasmolytic method or nanoindentation atomic force microscopy indicate that ethylene or ACC, as the biosynthetic precursor of ethylene, decreases the cell wall extensibility of stems [4,5]. This study aimed to determine the mechanism by which ACC treatment of azuki bean seedlings decreases the cell wall extensibility. First, the effect of ACC on the cell wall extensibility was confirmed by load–extension analysis using a tensile tester. ACC treatment decreased the total extensibility, when ACC suppressed the elongation growth and promoted the lateral growth of epicotyls (Figure 1A,B and Figure 2A). In the analysis of the cell wall extensibility using a tensile tester, it is affected by the cross-sectional area of the stem. Therefore, we corrected the total extensibility values by the cross-sectional area to eliminate its effect. Consequently, ACC decreased the total extensibility, although it was corrected for cross-sectional area (Figure 2B). The cell wall extensibility is a growth-limiting factor affecting the elongation growth of plants [3]. Collectively, these findings suggest that ACC suppresses the elongation growth of azuki bean epicotyls by decreasing the cell wall extensibility.

The chemical and physical properties of cell wall polysaccharides alter the mechanical properties of cell walls [18]. The irreversible (plastic) extensibility of the cell walls decreased by ACC treatment (Figure 2F). Changes in irreversible extensibility indicate changes in the viscous state of the cell walls. In addition, polymers with low molecular mass produce solutions of low viscosity, whereas high ones produce high viscosity [18]. In flowering plants with a Type I cell wall, the molecular mass of the xyloglucans is a major parameter that determines the viscosity of the cell wall. Based on previous reports, cell wall extensibility decreased when the molecular mass of xyloglucans increased, whereas it increased when the molecular mass of xyloglucans decreased [7,8,9,19]. In this study, ACC increased the molecular mass of xyloglucans (Figure 3). Thus, the increase in the molecular mass of xyloglucans is involved in the decrease in irreversible extensibility induced by ACC treatment. Wu et al. (2020) [5] reported that the molecular mass of pectic polysaccharides was increased in ACC-treated Arabidopsis hypocotyls. On the contrary, in azuki bean epicotyls, the elution pattern of pectic polysaccharides was not affected by ACC treatment (data not shown). Collectively, the molecular mass of cell wall polysaccharides, which varies among plant species, may be increased by ACC, and the increased molecular mass of polysaccharides may decrease the cell wall extensibility.

ACC increased the molecular mass of xyloglucans, indicating that the metabolic turnover of xyloglucans is affected by ACC. Figure 4 shows that ACC decreased the xyloglucan-degrading activity. The xyloglucan-degrading activity decreased under hypergravity conditions but increased under microgravity conditions in space [9,19]. On the contrary, the molecular mass of xyloglucans was increased by hypergravity but decreased by microgravity. Hence, changes in the activity of xyloglucan-degrading enzymes are considered important in the changes in the molecular mass of xyloglucans under different gravitational conditions. Therefore, the increase in the molecular mass of xyloglucans is due to ACC via the decrease in the xyloglucan-degrading activity.

The effects of ACC on the expression level of *VaXTHS4*, *VaXTH1*, and *VaXTH2* in the epicotyl region were examined (Figure 5). The results showed that of the three *XTH* genes, only *VaXTHS4* expression was regulated in response to ACC. Tabuchi et al. (2001) [13] reported that the VaXTHS4 protein shows only hydrolase activity and not any transglucosylase activity. In addition, Kaku et al. (2002) [20] indicated that treatment with the VaXTHS4 protein increased the extensibility of cell walls in azuki bean epicotyls. These findings indicate that ACC reduces xyloglucan-degrading activity by suppressing the expression of *VaXTHS4*, which may be necessary to increase the molecular mass of xyloglucans, thereby decreasing the cell wall extensibility and growth suppression. Markakis et al. (2012) [21] screened genes whose expression was regulated by ACC in Arabidopsis roots. *XTH21*, expansin (*EXP17*) and β-expansin (*EXPB3*) were included in the top 10 genes with the largest decrease in expression. The major activity of most XTHs is transglucosylase, although a few exhibits hydrolase activity [22]. XTH21, whose expression is downregulated by ACC in Arabidopsis, might be responsible for transglucosylase activity. In this study, we found that of the three *XTH* genes in azuki bean, *VaXTHS4* expression, which only exhibits hydrolase activity, was downregulated by ACC (Figure 5). Overexpression of XTH18, XTH19, and XTH20, which might be responsible for transglucosylase activity, modified hypocotyl growth and cell wall extensibility in Arabidopsis [23]. Multigene families of XTHs have been identified in various plants [24]. In addition to the three XTHs that we analyzed, other XTHs may be found in azuki bean. Changes in the gene expression have been reported in various members of XTHs in response to abiotic stresses that are thought to be mediated by ethylene [16]. It is also known that gene expression of various members of XTHs is changed by other phytohormones such as auxin [24]. Therefore, other XTHs, including XTHs with primary transglucosylase activity, may also be required for the modification of the molecular mass of xyloglucans by ACC. Moreover, another group of proteins in plant cell walls, namely, expansins, loosens the cell walls. The expression of expansin (*EXP17*) and β-expansin (*EXPB3*) was downregulated by ACC [21]. Therefore, expansins may also be associated with the regulation of the cell wall extensibility by ACC.

Table 1 summarizes the relationship between elongation growth, cell wall extensibility, and molecular mass of xyloglucans. The results of this study indicate that the increase in the molecular mass of xyloglucans by ACC, a precursor of the phytohormone ethylene, is responsible for the decrease in the extensibility of cell walls. On the contrary, the phytohormone auxin increases the extensibility of cell walls by decreasing the molecular mass of xyloglucans [7]. The molecular mass of xyloglucans was also affected by environmental factors such as light and gravity. Light irradiation decreased the extensibility of cell walls by increasing the molecular mass of xyloglucans [8]. Hypergravity decreased the extensibility of cell walls by increasing the molecular mass of xyloglucans, whereas microgravity increased the extensibility of cell walls by decreasing the molecular mass of xyloglucans [9,19]. Collectively, cell wall extensibility decreased when the molecular mass of xyloglucans increased, whereas it increased when the molecular mass of xyloglucans decreased. These findings suggest that in the regulation of stem elongation by various factors, changes in the molecular mass of xyloglucans modify cell wall extensibility, thereby regulating elongation growth in stems.

Exogenous ethylene or ACC application to plants not only inhibits the elongation growth, but also promotes the lateral expansion of stems. In this study, we showed that the decrease in the cell wall extensibility in the longitudinal direction via the increase in the molecular mass of xyloglucans is necessary for the ACC-induced inhibition of elongation growth in azuki bean epicotyls (Figure 1, Figure 2 and Figure 3). Based on previous reports, changes in the orientation of cortical microtubules promoted lateral expansion; the reorientation of cortical microtubules from transverse to longitudinal directions is induced when exogenous ethylene or ACC is applied to plants [17,25]. Moreover, microtubule-associated proteins (MAPs) such as γ-tubulin complex proteins, katanin, MAP65, and WAVE-DAMPENED2-LIKE5 (WDL5) played a role in the ACC-induced reorientation of cortical microtubules [17,26,27]. Collectively, when seedlings were treated with ethylene or ACC, the decrease in the cell wall extensibility in the longitudinal direction and the reorientation of cortical microtubules from transverse to longitudinal directions were induced, which resulted in short and thickened stems.

## 4. Materials and Methods

### 4.1. Plant Materials and Growth Experiments

Plant materials and growth experiments were prepared as previously described [17]. Etiolated seedlings of azuki bean (*Vigna angularis* (Wild.) Ohwi et Ohashi cv. Erimowase) with 25–30 mm long epicotyls were picked and marked with India ink in the 5 mm subhook region (3–8 mm below the hook). Marked seedlings were subjected to treatment with or without 10^−5^ *M* ACC for 5 h at 25 °C in dark conditions. After incubation, the length, diameter, and fresh weight of the marked regions were determined. “Ini. (initial)” represents the result immediately before treatment (0 h), “Cont. (control)” represents the result without ACC treatment for 5 h, and “ACC” represents the result with ACC treatment for 5 h. We performed all manipulations under dim green light (ca. 0.09 µmol m^−2^ s^−1^ at the handling level).

### 4.2. Cell Wall Extensibility

Epicotyl segments were prepared to measure the cell wall extensibility using a tensile tester (Tensilon RTM-25, Toyo Baldwin, Tokyo, Japan) as described previously [19]. Segments were clamped at a distance of 2 mm and stretched at 20 mm min^−1^ until a load of 10 g was generated. The total extensibility of cell walls was measured at the first load–extension curve just before the load of 10 g was generated, whereas the reversible extensibility was measured at the second load-extension curve. We calculated the irreversible extensibility as the difference between the slopes of the two extensions.

### 4.3. Molecular Mass of Xyloglucans

The molecular mass of xyloglucans in the hemicellulose-II fraction was measured as previously described [9]. The molecular mass distribution of xyloglucans was determined from the elution profiles of xyloglucans measured by iodine staining on a gel permeation column (TSK-GEL 5000 PW, Tosoh, Japan). The weight-average molecular mass of xyloglucans was calculated from the previously reported formula [7] using 10, 40, 70, 120, and 500 kDa of dextrans (Sigma, St. Louis, MO, USA) as molecular mass markers.

### 4.4. Xyloglucan-Degrading Activity

The extraction of crude enzyme and assay of xyloglucan-degrading activity was performed using the previously reported method [9]. The reaction mixture consisting of ca. 5 µg of cell wall proteins extracted with 1 *M* of NaCl from the cell walls and 20 µg of purified azuki xyloglucans (400–600 kDa) in 50 µL of 100 mM of sodium phosphate buffer (pH 6.0) was reacted at 37 °C for 6 h. The activity of xyloglucan-degrading enzymes was measured by iodine staining and indicated by the decrease in absorbance at 640 nm of the xyloglucan–iodine complex. The protein content in the enzymatic extract was determined using a protein assay kit (Bio-Rad, Hercules, CA, USA).

### 4.5. Gene Expression of XTHs

Quantitative RT–PCR was performed using the previously reported method [14]. In each treatment, gene expression was analyzed for three sets of six epicotyl segments as one sample. The primers contained the following sequences: forward 5′-GGGCTACCATTAAGCTTCAACCT-3′ and reverse 5′-TGTCAATTTCATCGTGGTTTCC-3′ for *VaXTHS4*, forward 5′-TGTTATCACTGGCTTCTGCTTCTT-3′ and reverse 5′-CAAAGGCCCAAGTAGGCACAT-3′ for *VaXTH1*, forward 5′-TCGCTAGCCTCTTCTGCACTCT-3′ and reverse 5′-TGTGATCGAAAGCCCATGTG-3′ for *VaXTH2*, and forward 5′-AGTCATCAGCTCGCGTTGAC-3′ and reverse 5′-TCAATCGGTAGGAGCGACG-3′ for 18S rRNA. All data were standardized against 18S rRNA, measured as an internal standard.

### 4.6. Statistical Analysis

The means and standard errors of the mean were calculated for individual measurement. The significance of the differences between control and ACC treatments was parsed using Student’s *t*-test.

## 5. Conclusions

ACC, the biosynthetic precursor of ethylene, may decrease the cell wall extensibility in azuki bean epicotyls via the following pathways: the suppression of xyloglucan-degrading activity via the downregulation of *VaXTHS4* expression, which only exhibits hydrolase activity, increases the molecular mass of xyloglucans, thereby decreasing the cell wall extensibility.

## Figures and Tables

**Table 1:** Relationship between elongation growth, cell wall extensibility, and molecular mass of xyloglucans.

Factor	Elongation Growth	Cell Wall Extensibility	Molecular Mass of Xyloglucans	Reference
ACC (ethylene)	Inhibition	Decrease	Increase	This study
Auxin	Promotion	Increase	Decrease	[7]
Light	Inhibition	Decrease	Increase	[8]
Microgravity	Promotion	Increase	Decrease	[19]
Hypergravity	Inhibition	Decrease	Increase	[9]

## Data Availability

The data and materials that support the findings of this study are available from the corresponding author upon reasonable request.
